# Elexacaftor-tezacaftor-ivacaftor in patients with cystic fibrosis ineligible for clinical trials: a 24-week observational study

**DOI:** 10.3389/fphar.2023.1178009

**Published:** 2023-06-02

**Authors:** Libor Fila, Alzbeta Grandcourtova, Alena Bilkova, Pavel Drevinek

**Affiliations:** ^1^ Department of Pneumology, Second Faculty of Medicine, Charles University and Motol University Hospital, Prague, Czechia; ^2^ Department of Medical Microbiology, Second Faculty of Medicine, Charles University and Motol University Hospital, Prague, Czechia

**Keywords:** cystic fibrosis, variant specific therapy, elexacaftor-tezacaftor-ivacaftor, lung function, nutritional status, sweat chloride concentration

## Abstract

**Introduction:** Seminal clinical trials with the triple combination of elexacaftor-tezacaftor-ivacaftor (ETI) demonstrated clinical efficacy in people with cystic fibrosis (pwCF) who carry at least one F508del mutation. However, due to exclusion criteria of these clinical trials, the effect of ETI was not studied in a substantial number of pwCF. Thus, we ran a single center trial to evaluate a clinical efficacy of ETI treatment in adult pwCF who were ineligible for enrollment in registration studies.

**Methods:** PwCF on ETI with prior lumacaftor-ivacaftor therapy, severe airway obstruction, well-preserved lung function, or with airway infection with pathogens at risk of more rapid decline in lung function formed the study group, while all the others on ETI formed the control group. Lung function, nutritional status and sweat chloride concentration were assessed before and after initialization of ETI therapy over a 6-month period.

**Results:** Approximately a half of the ETI-treated pwCF at the adult Prague CF center (49 of 96) were assigned to the study group. Their mean changes in body mass index ( + 1.04 kg/m^2^) and in sweat chloride concentration (−48.4 mmol/L) were similar to the control group ( + 1.02 kg/m^2^; −49.7 mmol/L), while the mean change in percent predicted forced expiratory volume in 1 s (ppFEV_1_; + 10.3 points) was significantly lower than in the control group ( + 15.8 points) (*p* = 0.0015). In the subgroup analysis, pwCF with severe airway obstruction (ppFEV_1_ <40) and pwCF with well-preserved lung function (ppFEV_1_ >90) showed a less potential for improvement in lung function during the ETI treatment than controls (median change in ppFEV_1_ + 4.9 points and + 9.5 points, respectively).

**Conclusion:** PwCF not eligible for inclusion in clinical trials demonstrated improvement in lung function and nutritional status following the initiation of treatment with the ETI combination. Moderate increase in ppFEV_1_ was observed in those with severe airway obstruction or well-preserved lung function.

## 1 Introduction

Cystic fibrosis (CF) is the most common inherited life-shortening disease among Caucasians. As indicated by a latest U.S. registry report (Cystic Fibrosis Foundation, 2022), life expectancy for people with CF (pwCF) is more than 50 years and the most frequent cause of death is progressive lung disease (Elborn, 2016).

For many decades, the cornerstone of CF care was only symptomatic approach. Mucoactive drugs along with chest physiotherapy, antibiotics, anti-inflammatory drugs, bronchodilators, oxygen therapy, noninvasive ventilation and ultimately lung transplantation have been used to treat impaired mucus clearance, airway infection and inflammation, and respiratory disorders (Girón Moreno et al., 2021).

The discovery of the cystic fibrosis transmembrane regulator (*CFTR*) gene in 1989 raised hopes for a curative therapy. Unfortunately, after more than 30 years the gene therapy is yet unavailable. However, since 2012 variant-specific therapy (VTS) has become a clinical reality. The first-in-class drug was ivacaftor (IVA), potentiating the CFTR protein function in pwCF carrying G551D or other gating mutations of the *CFTR* gene (Ramsey et al., 2011). IVA alone was followed by lumacaftor (in therapeutic combination with ivacaftor; LUM/IVA) for patients homozygous for F508del and tezacaftor (in combination with ivacaftor; TEZ/IVA) for patients homozygous for F508del or compound heterozygotes with residual function mutation (Wainwright et al., 2015; Rowe et al., 2017; Taylor-Cousar et al., 2017).

Finally, elexacaftor in combination with tezacaftor and ivacaftor (ETI) was approved for clinical use in 2019 for pwCF aged 12 years and older who carry at least one F508del mutation (Heijerman et al., 2019; Middleton et al., 2019). Subsequently, the ETI combination was shown to be superior to IVA for pwCF with the F508del along with the gating mutation, as well as to the TEZ/IVA combination for pwCF with the F508del along with the residual function mutation (Barry et al., 2021).

The above-mentioned studies demonstrated an improvement in lung function, nutritional status and quality of life together with a reduction in the pulmonary exacerbation rate and, in case of IVA and the ETI, the studies also proved a considerable decrease of sweat chloride concentration.

In general, inclusion and exclusion criteria of the respective clinical trials allowed only a clinically stable patient population to be enrolled. Thus, pwCF with severe airway obstruction (a percent predicted forced expiratory volume in one second (ppFEV_1_) below 40) or those harboring bacteria with a high risk of a rapid decline in lung function (*Burkholderia cenocepacia*, *Burkholderia dolosa*, *Mycobacterium abscessus*) were excluded from the trials. Also, pwCF with a well-preserved lung function (ppFEV_1_ above 90) were outside the range of 40–90, a key inclusion criterion that best sets the baseline for the change to observe statistically meaningful changes of ppFEV_1_ during the trial.

The aim of our study was to evaluate the real-world results of ETI treatment in pwCF who do not fulfill the classical criteria for participation in the trials. We also analyzed the clinical effect of the switch to ETI from LUM/IVA, which in contrast to TEZ/IVA switch (Heijerman et al., 2019) has not been studied before.

## 2 Materials and methods

### 2.1 Study participants

Adult pwCF attending the CF Center in Prague, Czech Republic, on ETI treatment were included in the study. The diagnosis of CF was confirmed in all cases by clinical presentation, sweat test and *CFTR* gene analysis. ETI treatment was initiated between July 2021 and April 2022. First 6 months of therapy represented a time period selected for the analysis of ppFEV_1_ and BMI on therapy (see below). Patients previously treated with LUM/IVA (n = 10), with ppFEV_1_ below 40 (n = 15) or above 90 (n = 20), or infected with *B. cenocepacia* (n = 14) or *M. abscessus* (n = 2) were all included in the study group (n = 49; 12 of them fell into more than one category), whereas others were controls (n = 46). Patients on ETI who were first treated with IVA or TEZ/IVA were not included in the study as they were, contrary to LUM/IVA treated pwCF, already assessed in registration studies.

### 2.2 Assessments

Demographic data (sex and age) and information regarding airway infection and previous VST were obtained from medical records. Lung function testing (spirometry) and assessment of nutritional status (measurement of body weight and body height with calculation of body mass index; BMI) were performed according to standard procedures during routine outpatient visits. The best values of ppFEV_1_ and BMI up to 6 months before (clinical appointments are performed every 3 months) and after initiation of ETI treatment (visits at month 1, 3, and 6) were recorded for the analysis. Sweat tests were performed using the Macroduct^®^ Sweat Collection System and the ChloroChek^®^ Chloridometer^®^ Sweat Chloride Analyzer (Wescor Inc., United States) before and after the initiation of ETI treatment (between month 3 and 6).

### 2.3 Statistical analysis

The changes in sweat chloride concentration values and in the best ppFEV_1_ and BMI values before and after the initiation of ETI treatment were compared between the study and control groups. Changes in best ppFEV1 values were also analyzed in subgroups of the study group. Statistical analysis was performed using TIBCO Statistica 13 (TIBCO Software Inc., USA). Normal distribution of the data was evaluated using Kolmogorov-Smirnov test and means (±SD) or medians (IQR) were used where appropriate, as well as t-tests and Mann-Whitney U-tests for comparison between groups. A *p*-value <0.05 was considered statistically significant.

## 3 Results

Demographic and clinical data of the patients on ETI are summarized in Table 1. In addition to *Pseudomonas aeruginosa*, *B. cenocepacia* and *M. abscessus*, several subjects had airway infections caused with methicillin-resistant *Staphylococcus aureus* (n = 6), *Achromobacter xylosoxidans* (n = 1), *Burkholderia stabilis* (n = 1) or *Burkholderia contaminans* (n = 1).

**TABLE 1:** Demographic and clinical characteristics of the patients.

Characteristics	Study group (n = 49)	Control group (n = 47)
Sex		
Female	23 (47%)	25 (53%)
Male	26 (53%)	22 (47%)
Age at the initiation of ETI treatment		
Mean (SD), years	29.2 (7.2)	29.4 (7.2)
CFTR gene mutation		
F508del/F508del	28 (57%)	31 (66%)
F508del/other:	21 (43%)	16 (34%)
CFTRdele2,3	6	6
1898+1G>A	2	0
2134delT	1	0
2184insA	1	0
2789+5G>A	1	0
3143del9	1	1
G27R	1	0
G542X	1	1
I336K	1	1
L1335P	1	0
N1303K	1	1
Q372X	1	0
R347P	1	0
W1282X	1	0
W57G	1	0
2176delA	0	1
574delA	0	1
622-1G>C	0	1
CFTRdel1-10	0	1
L1324P	0	1
L138ins	0	1
Airway infection		
*P. aeruginosa*†	11 (22%)	27 (57%)
*B. cenocepacia*	14 (29%)	0
*M. abscessus*	2 (4%)	0
CFTR modulator therapy before ETI		
Lumacaftor/ivacaftor	10 (20%)	0
FEV_1_		
Mean (SD), % predicted value	68.8 (30.4)	69.0 (14.1)
Distribution of FEV_1_ values before ETI		
>90% pred.	20 (41%)	0
≥40 to ≤90% pred.	14 (28%)	47 (100%)
<40% pred.	15 (31%)	0
Body mass index		
Mean (SD), kg/m^2^	23.21 (4.30)	21.74 (2.70)
Sweat chloride concentration before ETI		
Mean (SD), mmol/L	98.0 (11.0)	100.0 (10.7)

Nominal data are n (%). CFTR, cystic fibrosis transmembrane conductance regulator. Forced expiratory volume in 1 s (FEV_1_) and body mass index values were the best values in the 6 months before the initiation of ETI, treatment. †Intermittent and chronic infections with *P. aeruginosa* were both reported together.

Changes in lung function, nutritional status and sweat chloride concentration in the study and control groups after initiation of ETI treatment are shown in Table 2. The study population reached such changes in nutritional status and sweat chloride concentration that were comparable to the control group (i.e., there were found no significant differences in changes of both parameters between the groups). The improvement of lung function in the control group was higher than in the study group. Further stratification by sex showed no statistically significant difference between males and females within each of the two groups for FEV_1_ or BMI (Table 2).

**TABLE 2:** The changes in lung function, nutritional status and sweat chlorides on ETI treatment.

Parameter	Study group	Control group	*p*-value^a^
Change in FEV_1_			
Mean (SD), % predicted value	+10.3 (7.8)	+15.8 (8.7)	0.0015
Females (F)	+10.9	+14.8	
Males (M)	+9.9	+17.0	
*p*-value^a^ (F vs. M)	0.66	0.40	
Change in body mass index			
Mean (SD), kg/m^2^	+1.04 (0.97)	+1.02 (0.67)	n.s.
Females (F)	+1.01	+0.92	
Males (M)	+1.05	+1.08	
*p*-value^a^ (F vs. M)	0.59	0.42	
Change in sweat chloride concentration			
Mean (SD), mmol/L	‒48.4 (18.9)	‒49.7 (16.1)	n.s.

FEV_1_ = forced expiratory volume in 1 s.

^a^ *t*-test. n.s = not significant.

Further analysis of the lung function change was based on subgrouping of the study group participants according to following parameters: prior treatment with LUM/IVA, baseline ppFEV1 values and airway infection with pathogens at risk of more rapid decline in ppFEV1. Results are summarized in Table 3.

**TABLE 3:** Analysis of change in lung function in subgroups of the study population.

Subgroup	Change in FEV_1_ median (IQR), % predicted value
Prior treatment with LUM/IVA (n = 10)	+8.6 (+7.3 to +13.0)
ppFEV_1_ < 40%, without prior LUM/IVA treatment (n = 10)	+4.9 (+1.9 to +8.2)^a^
ppFEV_1_ > 90%, without prior LUM/IVA treatment (n = 19)	+9.5 (+4.4 to +14.3)^b^
ppFEV_1_ 40%–90% with pathogens at risk of more rapid FEV_1_ decline, without prior LUM/IVA treatment (n = 10)	+18.7 (+9.0 to +21.6)
Control group (n = 47)	+13.3 (+9.2 to +22.3)

ppFEV_1_ = a percent predicted forced expiratory volume in 1 s. IQR, interquartile range; LUM/IVA, lumacaftor/ivacaftor.

^a^ Lower than in the control group (*p* = 0.0003, Mann-Whitney U test);

^b^ lower than in the control group (*p* = 0.0067, Mann-Whitney U test).

**FIGURE 1:**
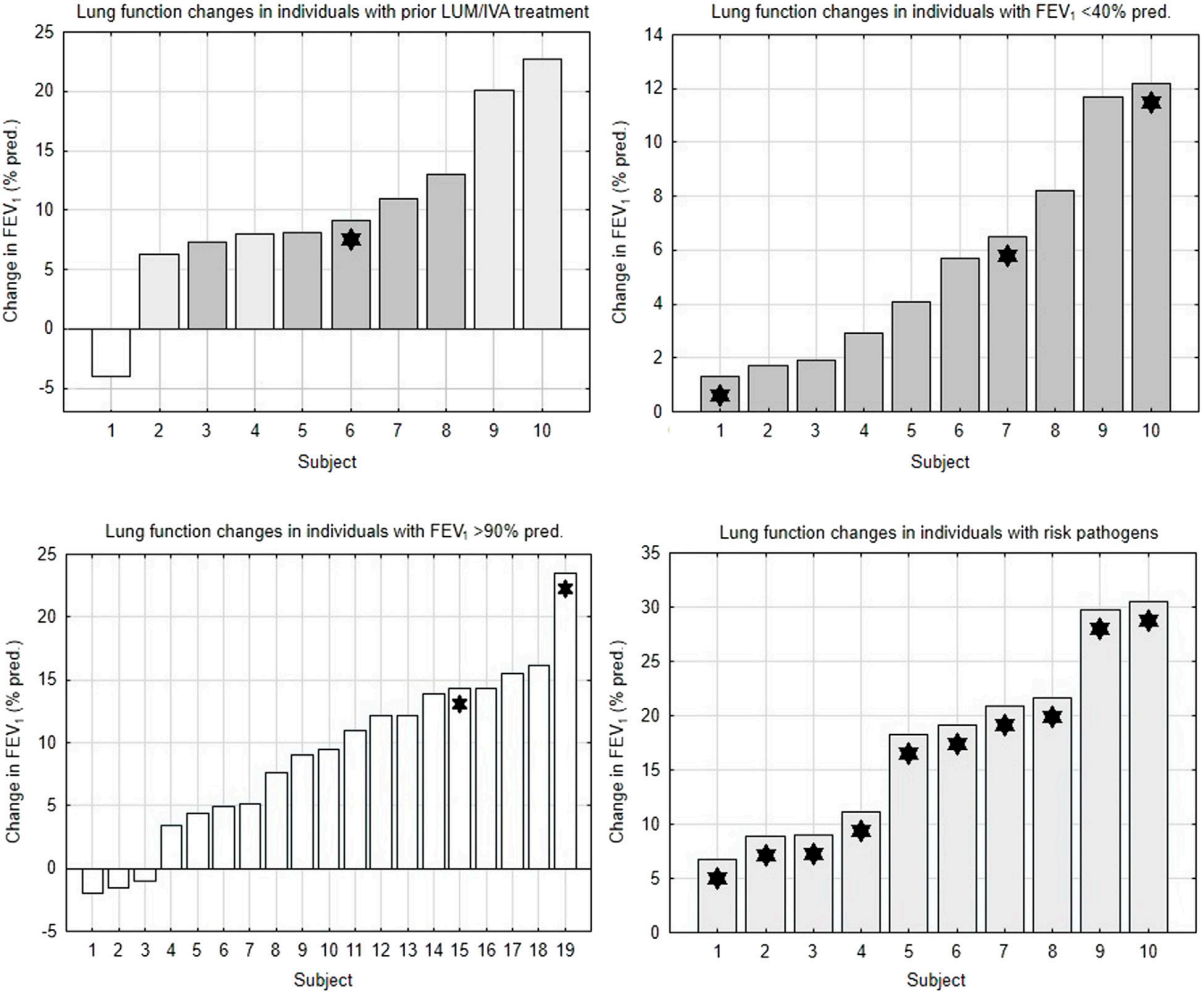
Changes in lung function in subgroups of the study population. FEV_1_ = forced expiratory volume in 1 s. LUM/IVA, lumacaftor/ivacaftor. White bars = subjects with FEV_1_ >90% pred. Light gray bars = subjects with FEV_1_ 40%–90% pred. Dark gray bars = subjects with FEV_1_ <40% pred. Asterisks = subjects with pathogens at risk of more rapid FEV_1_ decline.

This analysis showed that patients with severe airway obstruction or with well-preserved lung function had a lower potential for improvement with ETI treatment compared to the control group. Changes in lung function in individual subjects of studied subgroups are depicted in Figure 1.

## 4 Discussion

PwCF ineligible for participation in clinical trials represent a substantial part of the CF population. This is well documented in this study, where approximately a half of all patients treated with ETI (i.e., 49 of 96 patients) met one classical exclusion criterion or more. The aim of our study was to check the ETI effect on patients whose clinical conditions fall outside the inclusion criteria of clinical trials that were represented with registration studies for ETI. We believe that such postmarketing data are of paramount importance as it ensures not only a CF community, but also regulatory agencies and healthcare payers that the ETI therapy is effective in pwCF who were ineligible for classical clinical trials. For instance, the efficacy of the ETI treatment on pwCF with *B. cenocepacia* is found to be highly relevant to the Czech CF care where the prevalence of the infection is much higher (13%) compared to other European countries (4%) (Orenti et al., 2022).

Clinical data on lung function and nutritional status were collected from routine outpatient visits. We reported the best values of ppFEV_1_ and BMI during the 6 months before and after the initiation of ETI treatment for evaluation of clinical efficacy. This approach to analyze the best ppFEV_1_ and BMI values was chosen to minimalize the effect of intra-individual visit-to-visit variability, and for ppFEV_1_ it was similar to the common strategy of four-week screening/run-in period in clinical trials to ensure a patient is clinically stable. This is a similar approach to the assessment of pulmonary exacerbation in pwCF, where the current ppFEV_1_ value is compared to the best value in last 6 months (Goss, 2019).

The control group consisted of pwCF on ETI treatment who did not meet the common exclusion criteria for clinical trials participation. Their improvement in lung function was similar to the results published in phase 3 clinical trials with the ETI treatment: ppFEV_1_ +15.8 points in our study (over one to 6 months period) vs. +14.3 points in Week 24 (pwCF heterozygous for F508del, ETI vs. placebo) (Middleton et al., 2019) or +10.0 points in Week 4 (pwCF homozygous for F508del, ETI vs. TEZ/IVA combination) (Heijerman et al., 2019). The even better results in our cohort can be explained by the inclusion also of pwCF heterozygous for F508del with a mutation on the other allele which is regarded mild, as opposed to minimal function mutations, included as the only qualifying *CFTR* mutations in the registration study. The improvement in BMI was very comparable: +1.02 kg/m^2^ (control group in our study) vs. +1.04 kg/m^2^ (Middleton et al., 2019).

It is of note that our study group consisted of a heterogeneous population: subjects with severe airway obstruction as well as well-preserved lung function, subjects with pathogens at risk of more rapid FEV_1_ decline and subjects with prior LUM/IVA therapy. Compared to the control group, this group as a whole showed less of improvement in lung function (i.e., +10.3 points), in contrast to a very comparable improvement in nutritional status and in a decrease in sweat chloride concentration. In the subgroup analysis, subjects with severe airway obstruction and those with well-preserved lung function showed lower potential for improvement in their lung function. Our results indicated the lung function improvement to a lesser extent than published for pwCF with advanced lung disease by Burgel et al. (Burgel et al., 2021) or O'Shea et al. (O'Shea et al., 2021). While the former publication reported an improvement in mean ppFEV_1_ in patients without prior CFTR modulator therapy by + 12 points after 3 months (along with a reduction in the need for long-term oxygen therapy and non-invasive ventilation), and the latter showed ppFEV_1_ +9 points after 26 days on ETI, we observed the change of median ppFEV_1_ to be +4.9 points only. However, our observed change is similar to the work of Djavid and coworkers (ppFEV_1_ +5.5 points after 1 month) (Djavid et al., 2021).

Data on pwCF with prior therapy with LUM/IVA combination and their switch to ETI have not been previously published. Our small group showed an improvement in lung function (ppFEV_1_ +8.6 points) which indicates that the switch from LUM/IVA to ETI results in a very comparable outcome like the switch from TEZ/IVA to ETI (Heijerman et al., 2019).

Improvement in nutritional status in the whole study group (BMI +1.04 kg/m^2^) as well as the reduction in sweat chloride concentration was similar to our control group as well as to the clinical trial study by Middleton et al. (Middleton et al., 2019).

In conclusion, pwCF not eligible for inclusion in clinical trials demonstrated improvement in lung function and in nutritional status after initiation of the treatment with the ETI combination, comparable to the population studied by respective clinical trials. Likewise, they manifested the decrease in sweat chloride concentration. Less improvement in FEV_1_ was observed in a subcategory of pwCF who presented with severe airway obstruction or well-preserved lung function.

## Data Availability

The raw data supporting the conclusion of this article will be made available by the authors, without undue reservation.
